# Association between physical and mental health-related quality of life and adverse outcomes; a retrospective cohort study of 5,272 Scottish adults

**DOI:** 10.1186/1471-2458-14-1197

**Published:** 2014-11-21

**Authors:** Zia Ul-Haq, Daniel F Mackay, Jill P Pell

**Affiliations:** Institute of Health & Wellbeing, University of Glasgow, 1 Lilybank Gardens, Glasgow, G12 8RZ UK; Institute of Public Health & Social Sciences (IPH & SS), Khyber Medical University, Peshawar, KPK Pakistan

**Keywords:** Health-related quality of life, HRQoL, Scottish health survey, Mortality, Adverse outcomes

## Abstract

**Background:**

Health-related quality of life (HRQoL) is associated with adverse outcomes in disease-specific populations. This study examines whether it is also independent predictor of incident cancer, coronary heart disease (CHD) and mortality in the general population.

**Methods:**

The records of adult participants in the Scottish Health Survey 2003 were linked with hospital admissions, cancer registrations and death certificates. Cox proportional hazard models were used to explore the associations between quintiles of physical and mental component summary score (PCS and MCS respectively) of the SF-12 and adverse outcomes. Higher quintiles of both PCS and MCS indicate better health status.

**Results:**

Among the 5,272 study participants, the mean PCS score was 49 (standard deviation (SD) 10.3). Participants were followed-up for a mean of 7.6 years. On survival analysis the lowest quintile of PCS was a strong predictor of all-cause death (hazard ratio (HR) 2.81, 95% CI 1.76, 4.49), incident cancer (HR 1.63, 95% CI 1.10, 2.42), and CHD events (HR 1.99, 95% CI 1.00, 3.96), compared to the highest quintile. This association was independent of adiposity and other confounders. The mean MCS score 52 (SD 8.8). MCS quintile was not associated with incident cancer and CHD, and the association between MCS and all-cause death (HR 1.33, 95% CI 1.01, 1.75) became non-significant after adjustment for adiposity.

**Conclusion:**

Physical HRQoL is a significant predictor of a range of adverse outcomes, even after adjustment for adiposity and other confounders. This study highlights the importance of perceived health in the general population.

**Electronic supplementary material:**

The online version of this article (doi:10.1186/1471-2458-14-1197) contains supplementary material, which is available to authorized users.

## Background

Studies have shown that overall health-related quality of life (HRQoL) is associated with adverse outcomes, such as hospitalisation and death, in several disease-specific populations including: patients receiving haemodialysis [1], and patients with diabetes [2], pulmonary diseases [3], coronary heart disease (CHD) [4], stroke [5], cancer [6], arthritis [7], and liver disease [8]. However, results have conflicted in relation to the associations with the physical and mental components of HRQoL. Some studies have shown that physical HRQoL is significantly associated with adverse outcomes [2], but others have reported no association [3]. Similar contradictory findings have been reported for mental HRQoL [9, 10]. There is a general paucity of studies that have examined the associations between overall, physical or mental HRQoL and adverse outcomes in the general population [11].

SF-12 is a validated and widely used tool for measuring the generic HRQoL [12]. It is a shorter version of SF-36 and takes only one third of the time to complete the SF-36 and is, therefore, used in many large surveys [13]. The 12 questions of SF-12 are combined to form summary scores for physical and mental HRQoL, called physical component summary (PCS) and mental component summary (MCS). These summary scores are closely correlated with those produced using the SF-36 [14].

In a recent cross-sectional study using UK Biobank participants, we demonstrated that both high and low levels of adiposity were associated with poor self-reported health [15]. This association persisted after adjustment for potential confounders and was consistent across a number of anthropometric measurements including: body mass index (BMI), waist circumference, waist to hip ratio and body fat percentage. In a retrospective cohort study of 20,000 Scottish adults with 17 years follow-up, we found that poor self-reported health (SRH) at baseline was an independent predictor of all-cause death, incident cancer, psychiatric hospitalisations and CHD events [16]. In contrast, there was no independent association between poor mental health (measured by GHQ-12) and these adverse outcomes.

There is an ongoing debate that if a single question such as, SRH is available and is consistently reported to be a reliable measure then why to use a lengthy and multiple item questionnaires such as SF-36 and SF-12. However, health status measured by SRH, GHQ-12 and different measures of HRQoL are not identical [17, 18]. The SRH has clear advantage of reducing burden on respondents, particularly when the researchers are only interested for a broader view of overall health rather than a detail assessment. Nonetheless, SRH is a simple and reliable measure but it is at the cost of a detailed assessment on the individual domains of subjective well-being [19]. A multi-item measure, such as SF-36 and SF-12 offer more precise and complete multi-dimensional information of the individual’s perception of their own health. Therefore, several indicators exist but they are not the same and should not be used interchangeably [18]. The focus of this study is the use of HRQoL as an indicator of future health outcomes.

Adiposity is significantly associated with reduced overall health-related quality of life (HRQoL), even in the absence of medical comorbidity [20]. Two recent meta-analyses showed a significant dose–response relationship between adiposity and poor physical HRQoL, whereas mental HRQoL was only reduced in the morbidly obese [21, 22]. Higher BMI was also associated with many adverse outcomes, including incidence of CHD and cancer, and all-cause mortality [23, 24]. Adiposity can act as a mediator in the association between HRQoL and adverse outcomes.

In this study we investigate whether physical and mental HRQoL (derived from the SF-12) were independent predictors of incident cancer, CHD events, and all-cause deaths, and whether the associations varied by sex and BMI among a large representative sample from the Scotland adult population, after adjusting for potential confounding factors including: demographic and life-style factors, socio-economic status, hypertension, diabetes and adiposity.

## Methods

### Data sources

We used an extract of data from the Scottish Health Survey (SHS) 2003. (http://www.scotland.gov.uk/Topics/Statistics/Browse/Health/scottish-health-survey). Unlike, the earlier two SHS’s, which were conducted in 1995 and 1998, the SHS 2003 had no age limitation and it is the only SHS which included the SF-12 questionnaire to measure the physical and mental HRQoL. The details of the SHS have been described previously [20, 25]. In brief, participants were interviewed face to face by trained staff who collected information on demographics (including age and sex), socio-economic status (including area of residence and level of education) and lifestyle behaviours (including smoking habits and alcohol consumption) as well as completing the SF-12 questionnaire. The data collectors also measured the weight and height of study participants. In a follow-up visit, a qualified nurse measured blood pressure. The overall response rate was around 60%. Furthermore, over 90% of SHS participants consented to passive follow-up via record linkage to the Scotland-wide routine administrative databases held by the Information Services Division including: admissions to acute hospital (Scottish Morbidity Record SMR01), cancer registrations (Scottish Morbidity Record SMR06) and death certificates [26]. The SMR data undergo regular quality assurance checks and have been shown to be 99% complete and 94% accurate [27]. The linkage provided follow-up data up to a censor date of 31 December 2011.

### Inclusion criteria and definitions

SF-12 questionnaires were completed by SHS participants aged ≥20 years. Therefore our study was restricted to this age-group. Participants with a history of cancer or CHD at the time of the baseline interview were excluded from the study. Study participants were categorized into 20–44, 45–64 and ≥65 years of age. BMI was categorized, using standardized cut-off points, into underweight (<18.5 kg/m^2^), normal weight (18.5-24.9 kg/m^2^), overweight (25.0-29.9 kg/m^2^), and obese (≥30 kg/m^2^). Obese was further categorized into class I (30.0-34.9 kg/m^2^), class II (35.0-39.9 kg/m^2^) and class III obese (≥40 kg/m^2^). The level of education was treated as four categories from level 1 (less than O level grade C) to level 4 (degree level or above). The Scottish Index of Multiple deprivation (SIMD) was used as the measure of socio-economic status. SIMD is a validated and widely used area-based measure of multiple deprivations and is derived from participants’ postcodes of residence. SIMD is calculated using 31 indicators across 6 domains: income, employment, housing, health, education, skills and training, and area based access to the services (http://www.scotland.gov.uk/Topics/Statistics/SIMD). Self-reported smoking status was categorized into never, previous or current, and alcohol consumption was categorized as never, previous, within limits (<21 units/week for men; <14 units/week for women) and excessive. Hypertension was defined as ≥140/90 mmHg or use of anti-hypertensive medication. Presence of diabetes was based on self-report of a physician diagnosis. PCS and MCS were calculated from the 12 responses to the SF-12 questionnaire. Higher scores indicate better physical and mental health status respectively. For the ease of interpretation, PCS and MCS score quintiles were used in the analyses. Cancer was defined using International Classification of Diseases-10 (ICD-10) codes C00-C97. CHD event was defined as death or hospitalisation due to CHD. The latter was defined as first hospitalisation using ICD-10 code I20-I25 in the primary position of diagnosis.

### Statistical analyses

The characteristics of participants by quintile of PCS and MCS were analysed using chi-square tests or chi-square tests for trend for binary and ordinal data respectively. Separate Cox proportional hazard models were used to examine the associations between PCS and MCS quintile and three outcomes: all-cause deaths, cancer registrations, and CHD events (hospitalisations or death). The highest quintile (best HRQoL) was used as the referent category. The models were first adjusted for age only (model 1), followed by further adjustment for sex, SIMD, education level, smoking status, alcohol consumption, medical comorbidity (hypertension and diabetes) (model 2), and finally BMI was added as a covariate (model 3). Global test was used to check the proportional-hazards assumption of our survival models. We were interested in exploring whether sex, BMI or lifestyle behaviours could modify the relationship between HRQoL and outcomes, and thus influence the assessment of health outcomes and mortality by using HRQoL. Therefore we tested for statistical interactions between HRQoL summary scores and sex, BMI and lifestyle behaviours. All statistical analyses were performed using Stata version 12.1 (StataCorp, College Station, Texas). Statistical significance was defined as p < 0.05.

### Ethical approval

Scottish Health Survey participants consented to passive follow-up via record linkage to the Scotland-wide routine administrative databases held by the Information Services Division including: admissions to acute hospital. The authors used secondary analyses of health survey data using an anonymised data extract.

## Results

Of the 5,272 participants, 2,889 (54.8%) were women, 1,392 (26.4%) were current smokers, 1,096 (20.8%) consumed excessive amounts of alcohol and 1,316 (25%) had either hypertension, diabetes or both (Additional file 1). Their mean age at recruitment was 50 years (SD 16 years). The mean BMI was 27.5 kg/m^2^ (SD 5.1 kg/m^2^); 59 (1.1%) were underweight, 1,689 (32%) normal-weight, 2,152 (40.8%) overweight, and 1,372 (26%) obese. Of the obese, 940 (17.8%) were class I, 297 (5.6%) were class II, and 135 (2.6%) were class III obese (Table 1). Participants were followed-up for a maximum of 8 years (mean 7.6 years), providing a total of 40,067.2 person years of follow-up. Incident events included 391 (7.4%) all-cause deaths, 368 (7.0%) cancer registrations, and 134 (2.5%) CHD hospitalisations or deaths.

**Table 1:** **Characteristics of the participants by physical and mental component summary score quintiles of the SF-12**

	Physical component quintile (score)						Mental component quintile (score)					
	1 (<42)	2 (42–50.9)	3 (51–54.9)	4 (55–56)	5 (>56)		1 (<47)	2 (47–52.9)	3 (53–55.9)	4 (56–58)	5 (>58)	
	N = 1,055	N = 1,054	N = 1,057	N = 1,068	N = 1,038		N = 1,055	N = 1,054	N = 1,057	N = 1,068	N = 1,038	
	N (%)	N (%)	N (%)	N (%)	N (%)	P value	N (%)	N (%)	N (%)	N (%)	N (%)	P value
**Body Mass Index**												
Underweight	24 (2.3)	4 (0.38)	9 (0.85)	11 (1.0)	11 (1.1)	<0.001	21 (2.0)	4 (0.4)	15 (1.4)	11 (1.0)	8 (0.8)	0.045
Normal-weight	251 (23.8)	287 (27.2)	330 (31.2)	358 (33.5)	463 (44.6)		338 (31.7)	365 (34.0)	379 (36.0)	358 (32.6)	249 (25.4)	
Overweight	369 (35.0)	416 (39.5)	445 (42.1)	496 (46.4)	426 (41.0)		391 (36.7)	435 (40.5)	441 (41.9)	484 (44.0)	401 (40.9)	
Obese	411 (39.0)	347 (32.9)	273 (25.8)	203 (19.0)	138 (13.3)		315 (29.6)	271 (25.2)	217 (20.6)	247 (22.5)	322 (32.9)	
Class I	261 (24.7)	222 (21.1)	202 (19.1)	148 (13.9)	107 (10.3)		184 (17.3)	190 (17.7)	162 (15.4)	171 (15.6)	233 (23.8)	
Class II	95 (9.0)	95 (9.0)	48 (4.5)	34 (3.2)	25 (2.4)		81 (7.6)	54 (5.0)	39 (3.7)	56 (5.1)	67 (6.8)	
Class III	55 (5.2)	30 (2.9)	23 (2.2)	21 (2.0)	6 (0.6)		50 (4.7)	27 (2.5)	16 (1.5)	20 (1.8)	22 (2.2)	
**Sex**												
Men	463 (43.9)	476 (45.2)	508 (48.1)	508 (47.6)	428 (41.2)	<0.001	419 (39.3)	448 (41.7)	488 (46.4)	532 (48.4)	496 (50.6)	<0.001
Women	592 (56.1)	578 (54.8)	549 (51.9)	560 (52.4)	610 (58.8)		646 (60.7)	627 (58.3)	564 (53.6)	568 (51.6)	484 (49.4)	
**Age (years)**												
20-44	201 (19.1)	404 (38.3)	507 (48.0)	527 (49.3)	573 ( 55.2)	<0.001	473 (44.4)	508 (47.3)	518 (49.2)	484 (44.0)	229 (23.4)	<0.001
45-64	419 (39.7)	400 (38.0)	395 (37.4)	403 (37.7)	389 (37.5)		409 (38.4)	422 (39.3)	386 (36.7)	416 (37.8)	373 (38.1)	
≥65	435 (41.2)	250 (23.7)	155 (14.7)	138 (12.9)	76 (7.3)		183 (17.2)	145 (13.5)	148 (14.1)	200 (18.2)	378 (38.6)	
**SIMD**												
1 (most deprived)	267 (25.3)	197 (18.7)	153 (14.5)	157 (14.5)	105 (10.1)	<0.001	246 (23.1)	182 (16.9)	154 (14.6)	145 (13.2)	152 (15.5)	<0.001
2	247 (23.4)	197 (18.7)	204 (19.3)	179 (16.8)	168 (16.8)		233 (21.9)	187 (17.4)	182 (17.3)	196 (17.8)	197 (20.1)	
3	238 (22.6)	254 (24.1)	257 (24.3)	220 (20.6)	253 (24.4)		253 (23.8)	235 (21.7)	234 (22.2)	253 (23.0)	247 (25.2)	
4	189 (17.9)	209 (19.8)	230 (21.8)	248 (23.2)	252 (24.3)		198 (18.6)	240 (22.3)	234 (22.2)	223 (20.3)	233 (23.8)	
5 (least deprived)	114 (10.8)	197 (18.7)	213 (20.2)	264 (24.7)	260 (25.1)		135 (12.7)	231 (21.5)	248 (23.6)	283 (25.7)	151 (15.4)	
**Education** ^**a**^												
Level 1	139 (13.2)	168 (15.9)	170 (16.1)	187 (17.5)	169 (16.3)	<0.001	170 (15.7)	185 (17.2)	175 (16.6)	166 (15.1)	137 (14.0)	0.015
Level 2	143 (13.6)	163 (15.5)	193 (18.3)	198 (18.5)	200 (19.3)		167 (15.7)	175 (16.3)	211 (20.1)	209 (19.0)	135 (13. 8)	
Level 2	32 (3.0)	77 (7.31)	78 (7.4)	92 (8.6)	93 (9.0)		167 (7.8)	175 (6.1)	211 (7.5)	209 (8.3)	135 (5.5)	
Level 4	151 (14.3)	210 (19.9)	290 (27.4)	314 (29.4)	400 (38.5)		224 (21.0)	321 (29.9)	326 (31.0)	289 (26.3)	205 (20.9)	
None of these	590 (55.9)	436 (41.4)	326 (30.8)	277 (25.9)	176 (17.0)		421 (39.5)	329 (30.6)	261 (24.8)	345 (31.4)	449 (45.8)	
**Smoking status**												
Never	361 (34.2)	441 (41.9)	447 (42.3)	526 (49.3)	541 (52.1)	<0.001	390 (36.6)	465 (43.3)	490 (46.6)	536 (48.7)	435 (44.4)	<0.001
Previous	387 (36.7)	317 (30.1)	296 (28.0)	306 (28.7)	258 (24.9)		268 (25.2)	311 (28.9)	318 (30.2)	319 (29.0)	348 (35.5)	
Current	307 (29.1)	296 (28.1)	314 (29.7)	236 (22.1)	239 (23.0)		407 (38.2)	299 (27.8)	244 (23.2)	245 (22.3)	197 (20.1)	
**Alcohol consumption**												
Never	92 (8.7)	53 (5.03)	36 (3.4)	39 (3.7)	32 (3.1)	<0.001	53 (5.0)	41 (3.8)	34 (3.2)	42 (3.8)	82 (8.4)	0.016
Previous	99 (9.4)	39 (3.7)	36 (3.4)	26 (2.4)	25 (2.4)		75 (7.0)	39 (3.6)	35 (3.3)	37 (3.4)	39 (4.0)	
Within limit	704 (66.7)	739 (70.1)	733 (69.4)	802 (75.1)	721 (69.5)		687 (64.5)	774 (72.0)	748 (71.1)	812 (73.8)	678 (69.2)	
Excessive	160 (15.2)	223 (21.2)	252 (23.8)	201 (18.8)	260 (25.1)		250 (23.5)	221 (20.6)	235 (22.3)	209 (19.0)	181 (18.5)	
**Medical comorbidity**												
No	576 (54.6)	725 (68.8)	829 (78.4)	886 (83.0)	940 (90.6)	<0.001	734 (68.9)	836 (77.8)	859 (81.7)	879 (79.9)	648 (66.1)	<0.001
Yes	479 (45.4)	329 (31.2)	228 (21.6)	182 (17.0)	98 (9.4)		331 (31.1)	239 (22.2)	193 (18.4)	221 (20.1)	332 (33.9)	

*P* values were determined by χ2 test; SIMD, Scottish index of multiple deprivations; ^a^1 (Lower than O level Grade C), 2 (O level or equivalent), 3 (A level/other below degree), 4 (Degree level or above).

Overall, the mean PCS score was 49 (SD 10.3). The lowest quintile equated to <42 and the highest to >56. Compared to the participants who were in the highest quintile of PCS (better physical HRQoL), those in the lowest quintile were older and more likely to be obese, male, socio-economically deprived, smoke and have hypertension or diabetes, but were less likely to consume excessive amounts of alcohol or have a higher degree (Table 1). Overall, the mean MCS score was 52 (SD 8.8). The lowest quintile equated to <47 and the highest quintile to >58. There was a U-shaped relationship between BMI, level of education and socio-economic deprivation and higher MCS score (Table 1). Compared to the participants who were in the highest quintile of MCS (better mental HRQoL), those in the lowest quintile had similar proportions of obese, hypertension or diabetes, not socio-economically deprived, and educated (Table 1). Unlike, PCS there was no dose–response relationship between age, BMI, deprivation and hypertension or diabetes and MCS.

**Figure 1:**
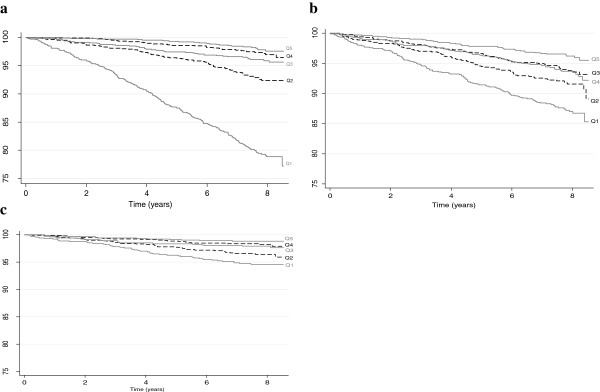
**Kaplan-Meier plot of the association between quintile of Physical Component Summary (PCS) of the SF-12 and adverse outcome. a**. All-cause death **b**. Cancer registration **c**. Coronary heart disease hospitalisation/death. Higher quintile indicate better perceived physical health; Q1 (worst), quintile 1 (PCS score <42); Q2, quintile 2 (PCS score 42 to 50.9); Q3, quintile 3 (PCS score 51 to 54.9); Q4 quintile 4 (PCS score 55 to 56); Q5 (best) quintile 5 (PCS score >56).

The Cox-proportional hazard models revealed that there were inverse dose–response relationships between baseline PCS and all-cause deaths, cancer registrations, and CHD events (Figure 1). Compared to those in the highest quintile (better physical HRQoL), participants in bottom two quintiles were significantly more likely to experience all-cause death, cancer registration and CHD events when adjusted for age only (Table 2). Further adjustment for sex, socioeconomic status, education level, smoking status, alcohol consumption, hypertension and diabetes attenuated the associations but the participants in the lowest quintile of PCS remained at significantly higher risk of adverse outcomes, compared to the highest quintile of PCS. When BMI was added to the model the lowest quintile of PCS remained a significant predictor of all-cause death (HR 2.64, 95% CI 1.76, 4.49), incident cancer (HR 1.63, 95% CI 1.10, 2.42), and CHD events (HR 1.99, 95% CI 1.00, 3.96), compared to the participants in the highest PCS quintile (PCS score >56) (Table 2). There was no significant interaction between PCS quintile and either sex (p = 0.968), BMI (p = 0.059), smoking (p = 0.069) or alcohol consumption (p = 0.328) in relation to any of the adverse outcomes.

**Table 2:** **Cox regression models of the association between quintiles of physical component summary score (PCS) of the SF-12 and adverse outcomes**

	Model 1		Model 2		Model 3	
	HR (95% CI)	P value	HR (95% CI)	P value	HR (95% CI)	P value
**All-cause death**						
PCS quintile						
1 (worst)	4.23 (2.70, 6.63)	<0.001	2.64 (1.66, 4.20)	<0.001	2.81 (1.76, 4.49)	<0.001
2	1.97 (1.22, 3.19)	0.005	1.44 (0.88, 2.34)	0.146	1.55 (0.95, 2.54)	0.078
3	1.45 (0.86, 2.42)	0.160	1.09 (0.65, 1.84)	0.747	1.15 (0.68, 1.94)	0.599
4	1.06 (0.61, 1.84)	0.826	0.90 (0.52, 1.57)	0.718	0.95 (0.55, 1.66)	0.869
5 (best)	1.00	-	1.00	-	1.00	-
**Cancer registration**						
PCS quintile						
1 (worst)	1.87 (1.29, 2.71)	0.001	1.60 (1.08, 2.37)	0.018	1.63 (1.10, 2.42)	0.015
2	1.58 (1.07, 2.31)	0.020	1.45 (0.98, 2.14)	0.067	1.47 (0.99, 2.19)	0.056
3	1.39 (0.93, 2.07)	0.110	1.29 (0.86, 1.93)	0.223	1.30 (0.86, 1.95)	0.212
4	1.50 (1.01, 2.23)	0.043	1.46 (0.98, 2.18)	0.062	1.45 (0.98, 2.17)	0.066
5 (best)	1.00	-	1.00	-	1.00	-
**Coronary heart disease hospitalisation/death**						
PCS quintile						
1 (worst)	2.57 (1.35, 4.93)	0.004	1.99 (1.01, 3.93)	0.047	1.99 (1.00, 3.96)	0.049
2	2.23 (1.15, 4.30)	0.017	1.82 (0.93, 3.59)	0.082	1.81 (0.92, 3.58)	0.087
3	1.61 (0.79, 3.25)	0.187	1.36 (0.67, 2.78)	0.398	1.36 (0.66, 2.78)	0.402
4	1.36 (0.66, 2.80)	0.405	1.19 (0.57, 2.47)	0.646	1.19 (0.57, 2.48)	0.640
5 (best)	1.00	-	1.00	-	1.00	-

Higher quintile indicate better physical health status; PCS, physical component summary quintile (score): 1 (<42), 2 (42 to 50.9), 3 (51 to 54.9), 4 (55 to 56), 5 (>56); HR, hazard ratio; CI, Confidence interval; Model 1 adjusted for age; Model 2 adjusted for age, sex, SIMD, education level, smoking status, alcohol consumption, hypertension and diabetes; Model 3 adjusted for same covariates as Model 2 plus body mass index.

There were inverse dose response relationships between the two lowest quintiles of MCS and all-cause death, but not with CHD events or cancer registration. Being in the lowest quintile of MCS was a significant predictor of all-cause death after adjustment for age (Table 3). When further adjusted for sex, socioeconomic status, education level, smoking status, alcohol consumption, hypertension and diabetes the hazard ratios were attenuated but remained statistically significant (HR 1.33, 95% CI 1.01, 1.75). When also adjusted for BMI, the association became statistically non-significant. There was no significant interaction between MCS and either sex (p = 0.062) or BMI (p = 0.767), or alcohol consumption (p = 0.367) in relation to any of the adverse outcomes.

**Table 3:** **Cox regression models of the association between quintiles of mental component summary score (MCS) of the SF-12 and adverse outcomes**

	Model 1		Model 2		Model 3	
	HR (95% CI)	P value	HR (95% CI)	P value	HR (95% CI)	P value
**All-cause death**						
MCS quintile						
1 (worst)	1.61 (1.24, 2.10)	<0.001	1.33 (1.01, 1.75)	0.041	1.25 (0.95, 1.65)	0.117
2	1.04 (0.76, 1.41)	0.824	1.00 (0.73, 1.37)	0.991	1.00 (0.73, 1.37)	0.998
3	0.83 (0.59, 1.15)	0.259	0.92 (0.66, 1.28)	0.603	0.88 (0.63, 1.23)	0.463
4	0.66 (0.47, 0.91)	0.012	0.77 (0.56, 1.08)	0.128	0.76 (0.55, 1.06)	0.104
5 (best)	1.00	-	1.00	-	1.00	-
**Cancer registration**						
MCS quintile						
1 (worst)	0.81 (0.59, 1.10)	0.171	0.75 (0.55, 1.03)	0.077	0.75 (0.55, 1.03)	0.079
2	0.75 (0.55, 1.03)	0.077	0.75 (0.54, 1.03)	0.075	0.74 (0.54, 1.03)	0.071
3	0.70 (0.51, 0.97)	0.033	0.73 (0.52, 1.01)	0.055	0.72 (0.52, 1.01)	0.054
4	0.78 (0.58, 1.06)	0.111	0.83 (0.61, 1.12)	0.215	0.82 (0.61, 1.11)	0.206
5 (best)	1.00	-	1.00	-	1.00	-
**Coronary heart disease hospitalisation/death**						
MCS quintile						
1 (worst)	0.91 (0.56, 1.48)	0.715	0.84 (0.51, 1.37)	0.480	0.84 (0.51, 1.38)	0.499
2	0.60 (0.35, 1.05)	0.072	0.61 (0.35, 1.06)	0.077	0.61 (0.35, 1.06)	0.082
3	0.55 (0.31, 0.97)	0.039	0.59 (0.33, 1.05)	0.071	0.59 (0.33, 1.05)	0.073
4	0.75 (0.46, 1.22)	0.252	0.79 (0.48, 1.29)	0.352	0.80 (0.49, 1.30)	0.363
5 (best)	1.00	-	1.00	-	1.00	-

Higher quintile indicate better mental health status; MCS, mental component summary quintile (score): 1 (<47), 2 (47 to 52.9), 3 (53 to 55.9), 4 (56 to 58), 5 (>58); HR, hazard ratio; CI, Confidence interval; Model 1 adjusted for age; Model 2 adjusted for age, sex, SIMD, education level, smoking status, alcohol consumption, hypertension and diabetes; Model 3 adjusted for same covariates as Model 2 plus body mass index.

## Discussion

Physical HRQoL was found to be a strong predictor of incident cancer, CHD events and all-cause mortality on follow-up. The associations were independent of adiposity and other potential confounders, and there was evidence of inverse dose–response relationships. In contrast, poor mental HRQoL was only a significant predictor of all-cause death and that was explained by adiposity. There were no statistically significant differences in the associations between men and women or by level of adiposity.

The majority of previous studies have focused on the association between HRQoL and mortality in diseases-specific populations and they have produced conflicting results. For example, in patients undergoing haemodialysis some studies have reported that both PCS and MCS were strong predictors of mortality [1]. Some reported that MCS was a significant predictor of mortality, but not PCS [10]. Others reported the reverse findings with PCS being a significant predictor of mortality, but not MCS [9]. In patients with pulmonary fibrosis, HRQoL did not predict death [3]. PCS was associated with higher mortality in diabetic patients, but not MCS [2]. In a study of patients with heart failure, MCS predicted mortality, but PCS did not [4]. Others have reported that both MCS and PCS were associated with higher mortality in atherosclerotic patients [5]. Similarly, only PCS was strongly associated with mortality in patients with rheumatoid arthritis [7], but both PCS and MCS were in patients with liver cirrhosis [8]. One recent study reported that HRQoL was the only psychosocial predictor of survival in cancer patients [6].

Very few studies have explored the association between HRQoL and adverse outcomes in the general population. In a recent German study, 4,259 participants, aged 20–79 years, completed the SF-12 at baseline and suffered 456 deaths over a mean of 9.7 years follow-up [11]. The Cox-proportional hazard models revealed that the lowest quartile of PCS was an independent predictor of mortality (fully adjusted HR 1.64, 95% CI 1.19, 2.27), compared to the highest quartile. In contrast, MCS was not a significant predictor of mortality (HR 0.97, 95% CI 0.74, 1.28). Other studies conducted in general population have been few in number and have focused mainly on people aged 60 years or more. A longitudinal study was conducted in Taiwan, in which 4,424 individuals, aged 65 years and older were followed over three years and 221 deaths recorded [28]. A 10-point decrease in both PCS and MCS scores was associated with higher mortality; relative risk (RR) 1.60, 95% CI 1.39, 1.83, and RR 1.16, 95% CI 1.01, 1.34, respectively. In a US study 2,166 participants, aged 65 years or older, completed SF-12 questionnaires at baseline and were passively followed-up over 28 months using data from their insurance records [29]. Participants in the lowest quartile of PCS had a higher risk of both all-cause deaths (HR 5.99, 95% CI 1.90, 18.95) and hospitalisation (HR 2.64, p < 0.001) than those in the highest quartile. Those in the lowest quartile for MCS were also at higher risk of death (HR 2.30, 95% CI 1.64, 3.22) and hospitalisation (HR 1.40, p < 0.001). A Spanish study followed 2,343 participants, aged 60 years and above for six years and recorded 212 deaths [30]. A five-point decrease in baseline PCS score was found to be a significant predictor of mortality (HR 1.28, 95% CI 1.17, 1.40) but this was not true for MCS (HR 1.05, 95% CI 0.97, 1.13). Our model 2 results are consistent with these previous studies.

Recently, in a larger study of the Scottish adult population, with follow-up of 17 years, we demonstrated that after full adjustment, poor baseline self-reported health was an independent predictor of all-cause death (HR 2.48 95% CI 2.16, 2.85), incident cancer (HR 1.32, 95% CI 1.09, 1.58), and CHD events (HR 2.26, 95% CI 1.79, 2.84) [16]. In contrast, after full adjustment, mental health (measured by GHQ-12) was not a significant predictor of these adverse outcomes. Similarly, in this study we showed independent associations between lowest quintile of baseline PCS (poor physical HRQoL) and all-cause death (HR 2.81, 95% CI 1.76, 4.49), incident cancer (HR 1.63, 95% CI 1.10, 2.42), and CHD events (HR 1.99, 95% CI 1.00, 3.96). In contrast, MCS was not associated with these adverse outcomes after adjustment for potential confounders.

We did not explore the underlying mechanism by which HRQoL may impact on morbidity and mortality. Self-perceived health is more inclusive and provides additional information by incorporating both objectively measured and subjective assumptions of health risk [31]. HRQoL may identify accurate health status by covering those aspects of health which are difficult to capture by objective measurements such as subclinical disease, help-seeking behaviour and health system [32]. Better HRQoL may reflect an individual’s attitude towards improving health, and thus adopting preventive measures. In contrast, the low HRQoL may result in neglecting of the primary or secondary prevention such as healthy diet, physical activity, screening and taking regular medication for existing medical conditions which may eventually result in early death or disease incidence [33]. MCS was not associated with health outcomes. The exact mechanism is not known but there was a dose–response relationship between base-line BMI, level of education, hypertension, diabetes and socio-economic deprivation and low PCS. In contrast, there was a U-shaped relationship with MCS. Our current findings that comorbidity, ageing, social and obesity gradient do not exist for MCS could partly explain the fact that the participants with low MCS has lower risk of cancer incidence and CVD events, compared to the high MCS.

We have previously conducted several studies on the association between adiposity and HRQoL [15, 20, 25] including two meta-analyses; one in children and adolescents [21] and the other in adults [22]. Collectively, these studies revealed that adiposity had a significant positive association with poor physical HRQoL with evidence of dose response. Adiposity is also associated with many non-communicable diseases, including CHD and cancers [34–36]. Recently, three large-scale meta-analyses have consistently reported that obesity is a significant predictor of mortality [37–39]. In this study we have shown that poor physical HRQoL is a strong independent predictor of all-cause death, cancer incidence and CHD events. In contrast mental HRQoL is a predictor of all-cause deaths but not independently of adiposity. It is possible that mental HRQoL and adiposity lie on the same causal pathway. It is not possible to be certain of the direction of effect. Poor mental HRQoL may impact on lifestyle and, therefore, increase the risk of adiposity. Conversely, adiposity may itself predispose to poor mental HRQoL.

Commonly, population health is measured in terms of morbidity and mortality. Our results further strengthen the growing evidence that perceived health provides additional information and is predictive of future morbidity and mortality. It should be considered when undertaking both individual and community health assessments. It has been suggested that perceived health may be a stronger predictor of adverse outcomes than many objective measures of health [33].

We used data from a large representative sample of Scottish general population, and adjusted our analyses for a series of potential confounders. The “representativeness” of a health survey is generally determined by the higher number of responses which is based on the updated and correct sampling frame, study design and non-responses. The Scottish health survey has rigorous methodology and maintains the overall higher response rate of 60% or above from the eligible households. The age and sex proportion of the adult respondents is externally validated with the General Registrar Office for Scotland mid-year population estimates. The Scottish health survey used weighting to take account of the underrepresentation of the large households responses and non-response biases. It is also considered to be representative of the Scottish population in terms of the SIMD quintiles [26]. BMI and blood pressure were measured by trained individuals using standard operating procedures. The presence of diabetes was self-reported but based on physician diagnoses. The SF-12 is a validated and very widely used measure of HRQoL in the general population [12]. The Scottish Morbidity Record (SMR) has pan-Scotland coverage undergoes regular quality assurance checks [27]. Use of a cohort design enabled us to demonstrate a temporal relationship between baseline PCS and MCS and incident disease, and all-cause mortality and thereby avoid reverse causation. The advantage of retrospective cohort study includes readily access to data but it is at the cost of lack of control over the data collection. By treating PCS and MCS as ordinal data we were able to examine whether there were evidence of a dose relationship. Previous studies have also used the quintiles of PCS and MCS for the ease of interpretation [11, 40] but it may lead to considerable power loss and misleading results. We have used the SF-12 PCS and MCS scoring system for the SF-12 but the alternative scoring systems are also available such as RAND-12 and multidimensional item response theory (MIRT) models [17, 41]. Previous studies suggested that these alternatives models may have more power to detect the true differences and can also provide more reliable and intuitive scoring, particularly when the SF-12 is used to assess the mental health status [17, 41].

## Conclusions

Poor physical health-related quality of life is a strong predictor of all-cause death, cancer incidence and CHD events. The association is independent of adiposity and other potential confounders. This study adds to the growing evidence that perceived health is an important predictor of health risk, independent of adiposity or comorbidity, and should be considered when assessing the health of individuals and communities.

## Electronic supplementary material

Additional file 1:**Characteristics of the participants (n=5,272).**(PDF 161 KB)

## Abbreviations

HRQoL: Health-related quality of life
CHD: Coronary heart disease
SHS: Scottish Health Survey
PCS: Physical component summary
MCS: Mental component summary
HR: Hazard ratio
CI: Confidence interval
BMI: Body mass index
NHS: National Health Service
SMR: Scottish Morbidity Record
SIMD: Scottish Index of Multiple deprivation.
