# Supplementary material for: Association between physical and mental health-related quality of life and adverse outcomes; a retrospective cohort study of 5,272 Scottish adults
Source: BMC Public Health. 2014 Nov 21;14:1197. doi: 10.1186/1471-2458-14-1197 (PMC4256892; doi:10.1186/1471-2458-14-1197)
Supplement: Supplementary file 1 — Additional file 1:Characteristics of the participants (n=5,272).(PDF 161 KB) [file 12889_2014_7330_MOESM1_ESM.pdf]

Table S1. Characteristics of the participants (n=5,272)

	N (%)
**Body Mass Index**	
Underweight	59 (1.1)
Normal-weight	1689 (32.0)
Overweight	2152 (40.8)
Obese	1372 (26.0)
Class I	940 (17.8)
Class II	297 (5.6)
Class III	135 (2.6)
**Sex**	
Men	2383 (45.2)
Women	2889 (54.8)
**Age (years)**	
20-44	2212 (42.0)
45-64	2006 (38.1)
≥65	1054 (20.0)
**SIMD**	
1 (most deprived)	879 (16.7)
2	995 (18.9)
3	1222 (23.2)
4	1128 (21.4)
5 (least deprived)	1048 (19.9)
**Education[a]**	
Level 1	833 (15.8)
Level 2	897 (17.0)
Level 2	372 (7.1)
Level 4	1365 (25.9)
None of these	1805 (34.2)
**Smoking status**	
Never	2316 (43.9)
Previous	1564 (29.7)
Current	1392 (26.4)
**Alcohol consumption**	
Never	252 (4.8)
Previous	225 (4.3)
Within limit	3699 (70.2)
Excessive	1096 (20.8)
**Medical comorbidity**	
No	4565 (86.6)
Yes	707 (13.4)
**PCS**	
Mean (SD)	49.0 (10.3)
**MCS**	
Mean (SD)	51.9 (8.8)

SIMD, Scottish index of multiple deprivations; [a]1 (Lower than O level Grade C), 2 (O level or equivalent), 3 (A level/other below degree), 4 (Degree level or above)
